# Dominance of the ST20 *stG62647* Lineage Among Invasive *Streptococcus dysgalactiae* subsp. *equisimilis* Infections in Toronto, Canada

**DOI:** 10.3390/microorganisms14040878

**Published:** 2026-04-14

**Authors:** Kayleigh Gauvin, Kevin Li, Fengyang Hsu, Allison McGeer, Nahuel Fittipaldi

**Affiliations:** 1 GREMIP and CRIPA, Department of Pathology and Microbiology, Faculty of Veterinary Medicine, University of Montreal, St-Hyacinthe, QC J2S 2M2, Canada; kayleighgauvin@gmail.com (K.G.); kevin.li.2@umontreal.ca (K.L.); fengyang.hsu@umontreal.ca (F.H.); 2 Department of Microbiology, Sinai Health System, Toronto, ON M5G 1X5, Canada; allison.mcgeer@sinaihealth.ca; 3 Department of Laboratory Medicine and Pathobiology, University of Toronto, Toronto, ON M5S 3K3, Canada

**Keywords:** *Streptococcus dysgalactiae* subsp. *equisimilis*, SDSE, invasive infection, ST20 lineage, *emm* typing, multilocus sequence typing, whole-genome sequencing, population genomics, antimicrobial resistance, Canada

## Abstract

*Streptococcus dysgalactiae* subsp. *equisimilis* (SDSE) is an emerging cause of invasive disease, yet contemporary genomic data from Canada remain scarce. We investigated 56 cases of invasive SDSE infection identified between 2018 and 2022 in two major tertiary care teaching hospitals in Toronto, Ontario, and characterized 49 corresponding isolates by whole-genome sequencing. Nearly three-quarters of infections were caused by the globally expanding ST20 *emm* type *stG62647* lineage. Patients infected with this lineage were significantly older than those infected with non-ST20 lineages across both bloodstream and non-blood infections. Core-genome phylogenetic analysis revealed a highly clonal ST20 cluster, although two isolates had divergent *emm* types suggesting recombination at the *emm* locus. Non-ST20 lineages were numerically smaller and genetically more heterogeneous, including distinct sublineages within ST3 and ST34. All isolates were susceptible to β-lactams and vancomycin. Resistance to tetracycline, erythromycin, and clindamycin was detected in a subset of isolates and was associated with genes *tetM*, *tetO*, *ermA*, *ermB*, and *msrD*. Several antimicrobial resistance determinants were located on mobile genetic elements, including integrative and conjugative elements. Our findings provide a contemporary genomic view of invasive SDSE in Toronto, highlighting the dominance of the ST20 *stG62647* lineage in agreement with recent global observations.

## 1. Introduction

*Streptococcus dysgalactiae* subsp. *equisimilis* (SDSE) is a Gram-positive pathogen that colonizes the upper respiratory, gastrointestinal, and genital tracts of otherwise healthy individuals, and can also cause a wide range of diseases, from pharyngitis and skin and soft-tissue infections to severe invasive conditions such as necrotizing fasciitis and streptococcal toxic shock syndrome [1,2,3].

SDSE is typically β-hemolytic, displaying Lancefield group C or G antigens, although group A and L isolates, as well as rare α-hemolytic variants, have also been reported [4,5]. Genetically, SDSE is closely related to *Streptococcus pyogenes* and shares many of its core virulence determinants [6,7,8,9]. For example, in both species, the M protein plays a central role in resistance to phagocytosis, and its encoding gene, *emm*, is the basis of the widely used *emm* typing scheme [8]. However, SDSE shows substantial genetic diversity due to frequent recombination and horizontal gene transfer [9,10], and can affect several animal species in addition to humans [1,8].

Human SDSE invasive infections are increasing worldwide, driven in part by the emergence of a novel multilocus sequence typing (MLST) sequence type (ST) 20 *emm* type *stG62647* clone [6,10,11,12,13,14]. A recent genomic study further confirms the presence and expansion of this lineage in North America [15]. In Canada, previous s work has characterized SDSE infections in Manitoba from 2007 to 2014 [16,17], but no recent data are available for subsequent years or for other areas of the country. Here we describe invasive SDSE cases identified during 2018–2022 in two major teaching hospitals in Toronto and characterize the genomic features of the associated isolates, thereby providing a contemporary genomic characterization of invasive SDSE in a large urban Canadian setting and enabling direct comparison with earlier regional data and recent international reports.

## 2. Materials and Methods

### 2.1. Isolate Collection and Clinical Data

Data for 56 cases of SDSE invasive infection identified between 1 January 2018, and 15 March 2022, were obtained from two tertiary-care hospitals in Toronto, Canada. A case of invasive SDSE infection was defined as the isolation of group C or G *Streptococcus*, with SDSE species confirmation by whole-genome sequencing (WGS, see below), from a normally sterile site (e.g., blood, cerebrospinal, pleural, peritoneal, pericardial, or joint or bursal fluids, and bone, abscess aspirate, or surgical tissue). Isolates were initially identified by β-hemolysis on sheep blood agar and carbohydrate antigen grouping [18]. Although both group C and group G isolates were targeted for inclusion, all recovered isolates belonged to group C. We aimed to include one isolate per case; however, only 49 isolates remained viable for further analysis (Table S1). The study was based on a predefined, de-identified dataset that included only minimal metadata (patient age, sex, and specimen source) associated with each isolate. Detailed clinical variables such as comorbidities, treatment, and outcomes were not available within the scope of this study.

### 2.2. Bacterial Growth, DNA Extraction, and Whole-Genome Sequencing

Bacteria were grown on Columbia agar plates supplemented with 5% defibrinated sheep-blood (BD, Mississauga, ON, Canada) at 37 °C under 5% CO_2_, or, for DNA preparations, in Todd-Hewitt broth (BD) supplemented with 2% yeast extract (Thermo Fisher Scientific, Saint-Laurent, QC, Canada). To prepare genomic DNA, harvested organisms were resuspended in TE buffer, pH 7.6, containing 10 U/µL mutanolysin (Millipore-Sigma Oakville, ON, Canada) and 100 mg/mL lysozyme (Thermo Fisher) and incubated for 2 h at 37 °C. DNA was then purified using the QIAamp DNA Blood Kit (QIAGEN, Toronto, ON, Canada) following the manufacturer’s instructions for Gram-positive bacteria. Genomic libraries were prepared at Génome Québec (Montreal, QC, Canada) with NEBNext Ultra II DNA Library Prep Kits (NEB, Whitby, ON, Canada) and sequenced as paired-end 150 bp reads on an Illumina NovaSeq 6000 instrument (Illumina, San Diego, CA, USA).

### 2.3. Genomic and In Silico Analyses

We used Kraken V2 [19] to confirm the taxonomic classification of the isolates as SDSE. To confirm Lancefield grouping, we used SRST2 v0.2.0 [20] to map short-read sequences to a custom database of genes encoding enzyme clusters involved in the expression of groups A, C, and G carbohydrate antigens, based on previously described sequence data for reference strains of these Lancefield groups [21]. *de novo* genome assemblies were generated with the A5-miseq pipeline v20160825 [22], and resulting contigs were annotated with Prokka v1.14.6 [23], using a custom annotation database derived from SDSE strain MGCS36044 (GenBank accession no. GCA_029234115.1) [10]. MLST profiles were obtained directly from the short-read sequencing data using SRST2 and the PubMLST database [24]. *emm* typing was performed using a previously described bioinformatics pipeline [25] and the *emm* database curated by the United States Centers for Disease Control and Prevention. Antimicrobial resistance (AMR) genes were identified using SRST2 and the CARD v3.0.8 database [26]. The presence of MGEs was evaluated using Abricate v1.4.0 (https://github.com/tseemann/abricate, accessed on 1 December 2025) with the PlasmidFinder database [27], and with ICEscreen v1.3.3 [28]. To screen for the presence of superantigen-encoding genes, we used BLASTn and previously described primers and amplicons for genes *speA*, *speC*, *speH*, *speJ*, *speL*, *speM* [29], *speF*, *speG*, *speI*, *speK*, *smeZ* [30], and *sdm* [31]. We used the same approach and sequences derived from the annotation of various SDSE reference genomes to screen for the presence of virulence factor-encoding genes *hasC*, *ska*, *slo*, *scpA*, *scpB, silB* and *sda*.

### 2.4. Phylogenetic Analyses

Core-genome-based phylogenetic analysis was performed as follows: For each isolate, single nucleotide polymorphisms (SNPs) were identified relative to the genome of the reference strain MGCS36044 using the Snippy v4.6.0 algorithm (https://github.com/tseemann/snippy, accessed on 1 December 2025). Then, the dataset was reduced to the subset of core-genome SNPs with Snippy-core. Maximum-likelihood phylogenetic trees were generated from the resulting SNP alignment with FastTree v2.1.10 [32] using the Shimodaira–Hasegawa (SH)-like local support method to assess branch reliability and visualized with the ggtree package in R [33]. For comparative purposes, we included the genomes of 57 well-characterized invasive isolates from previously published studies, selected to represent major SDSE genetic lineages, *emm* types, and geographic regions (Canada, Australia, China, France, Japan, Norway, Spain, the United Kingdom, and the United States), with priority given to isolates with available metadata (Table S2).

### 2.5. Antimicrobial Susceptibility Testing

Susceptibility to penicillin G, vancomycin, tetracycline, gentamicin, clindamycin, and erythromycin was assessed by the Kirby–Bauer disk-diffusion method [34] on Mueller-Hinton agar supplemented with 5% defibrinated sheep blood. The D-test was used to detect inducible resistance to clindamycin. Zone diameters were interpreted according to CLSI guidelines for β-hemolytic streptococci [35]. The following interpretive breakpoints were used: isolates were considered susceptible when inhibition zones were ≥24 mm for penicillin G (10 U), ≥17 mm for vancomycin (30 µg), ≥23 mm for tetracycline (30 µg), ≥21 mm for erythromycin (15 µg), and ≥19 mm for clindamycin (2 µg). Gentamicin susceptibility was interpreted using *S. aureus* breakpoints, as no CLSI criteria are established for β-hemolytic streptococci [35].

### 2.6. Statistical Analysis

Associations between patient or infection characteristics (age group, sex, infection source, and year of infection) were assessed using χ^2^, Fisher’s exact, or Wilcoxon rank-sum (Mann–Whitney U) tests, as appropriate. Relationships between *emm* types and STs were evaluated using χ^2^ tests, and concordance between typing schemes was quantified with the Wallace coefficient.

## 3. Results

### 3.1. Invasive SDSE in Toronto Mostly Affects Older Adults and Commonly Presents as Bloodstream Infection

To define the patient populations most affected, and to identify potential relevant epidemiologic patterns, we first characterized the demographic context of 56 invasive SDSE infections identified in two adult teaching hospitals in Toronto, Canada. Among the 56 cases the median patient age was 65 years (range 0 days–97 years). One case occurred in a newborn, 19 (34%) in adults 18–59 years, 22 (39%) in adults 60–74 years, and 14 (25%) in adults ≥75 years (Table 1).

### 3.2. Laboratory Identification and Whole-Genome Sequencing Confirmed All Isolates as SDSE Expressing Lancefield Group C Antigen

Hospital microbiology laboratories initially identified the isolates recovered from the 56 patients as group C *Streptococcus* based on characteristic β-hemolysis on sheep blood agar and latex agglutination for Lancefield grouping [18]. To ensure accurate classification, we performed WGS and used Kraken V2 [19] to speciate the isolates. Only 49 of 56 isolates remained viable and the analysis confirmed that all 49 belonged to SDSE. Examination of the sequencing data indicated that 46–51% of reads were classified within the *S. dysgalactiae* group and 15–21% were specifically assigned to SDSE (Table S1). A small proportion (3–4%) of reads mapped to *S. dysgalactiae* subsp. *dysgalactiae* or only to the genus *Streptococcus*, a pattern typical of Kraken assignments among closely related taxa. To verify Lancefield phenotypic results, we next examined the genome assemblies for genes encoding the enzymes responsible for the Lancefield carbohydrate antigen biosynthesis. All 49 isolates possessed a Lancefield locus consistent with group C antigen expression.

To assess the possibility of selection bias, we compared the seven non-sequenced cases with the 49 sequenced cases using the available variables summarized in Table S1. The non-sequenced cases included four female and three male patients, with ages of 0, 33, 34, 38, 39, 65, and 68 years; excluding the neonatal case, the remaining six had a median age of approximately 39 years (range 33–68). Four of the seven non-sequenced cases were bloodstream infections, and three were from non-blood sterile sites (two operating room/tissue specimens and one abscess). These cases were distributed across the study period from 2019 to 2021, without evidence of temporal clustering. Given the small number and heterogeneity of these cases, this comparison is descriptive in nature. However, no consistent pattern was identified across the available variables to suggest that the non-sequenced cases represented a distinct subgroup or a major source of bias in the subset of isolates included in the genomic analyses.

### 3.3. Dominance of the ST20–stG62647 Lineage and a Conserved Virulence Gene Repertoire Across SDSE Isolates

**Figure 1:**
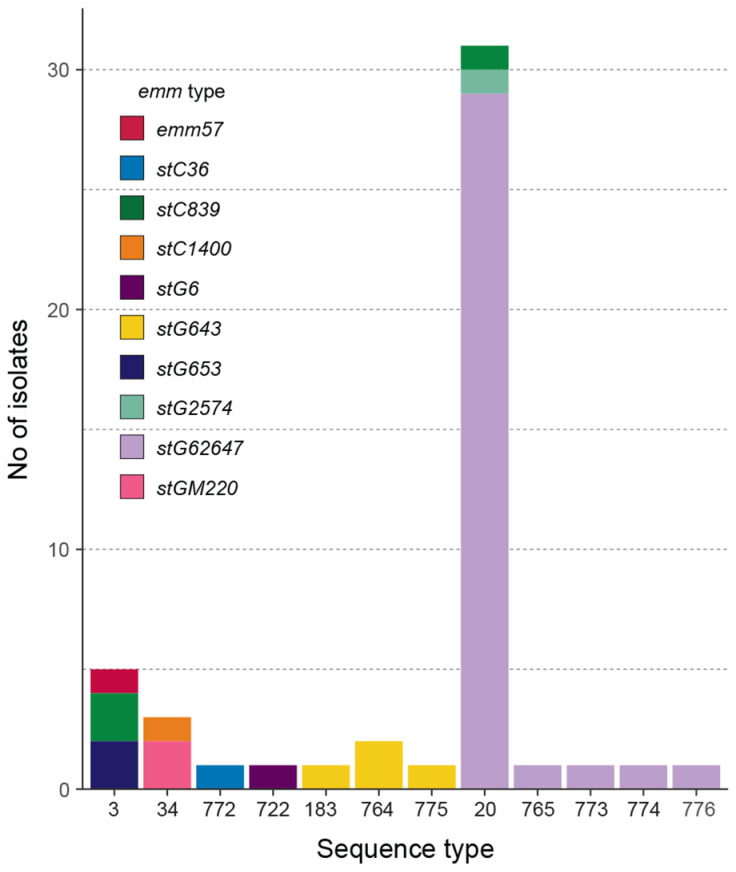
Association of multilocus sequence typing (MLST) sequence types (STs) with *emm* types among *Streptococcus dysgalactiae* subsp. *equisimilis* isolates. Bars show the number of isolates within each ST, with colors representing distinct *emm* types. The most prevalent ST was ST20 (31 isolates), and the most frequent *emm* type was *stG62647* (33 isolates). The combination ST20–*stG62647* accounted for 29 isolates, representing the dominant genotype in the collection.

To begin to define the population structure of invasive SDSE in Toronto and identify lineage-specific genomic traits, we further characterized the isolates using *emm* typing, MLST, and virulence-gene profiling. *emm* types and STs were determined in silico from assembled genomes; virulence and superantigen genes were identified by comparison to curated databases. Thirty-three isolates were *emm* type *stG62647*, which dominated the population. Other *emm* types included *stG643* (*n* = 4), *stC839* (*n* = 3), *stG653* (*n* = 2), *stGM220* (*n* = 2), and one isolate each of *emm57*, *stC1400*, *stC36*, *stG2574*, and *stG6*. In silico MLST of the 49 isolates identified 12 STs, seven of which were novel (Table S3). There was a clear dominance of ST20 (*n* = 31) and closely related derivatives ST765, ST773, ST774, and ST776 (*n* = 1 each). Five isolates belonged to ST3, three to ST34, two to ST764, and one each to ST183, ST775, ST722, and ST772. The relationships between MLST and *emm* types are shown in Figure 1. *emm* types often correlated with specific STs or clonal complexes (CCs), indicating a high likelihood that strains within the same CC share an *emm* type (*emm* type vs. CC, Wallace coefficient = 0.833, 95% CI 0.669–0.998). However, some variability remained (CC vs. *emm* type, W = 0.780, 95% CI 0.614–0.958).

We next screened the genomes for superantigen and selected virulence factor genes. *speG* was present in 42 (86%) of 49 isolates, whereas none of the other 12 tested superantigen genes were detected (Table S4). The virulence genes *ska* (streptokinase) and *slo* (streptolysin O) were present in all isolates. The gene *hasC*, which encodes a UDP-glucose dehydrogenase, was also universally detected but genes *hasAB* were not found. An IS1548 insertion disrupting *silB*, a regulatory gene within the *sil* locus that modulates virulence gene expression, was detected in all ST20 isolates and their derivatives (ST765, ST773, ST774, and ST776), whereas all but one non-ST20 isolates carried an intact *silB* gene; the complete *sil* locus was absent from the genome of that single non-ST20 isolate. *scpA*, encoding a serine protease, was highly prevalent (44/49, 90%). In contrast, *scpB*, encoding a second serine protease, was present in only five (10%) isolates, all of which carried an intact *silB* gene, whereas *sda*, encoding a streptodornase, was found in four (8%) isolates (Table S4).

### 3.4. Core-Genome-Based Phylogenetic Analysis Shows a Homogeneous ST20-stG62647 Lineage with Divergent emm Recombining Sublineages and Relatively High Diversity in Non-ST20 Lineages

**Figure 2:**
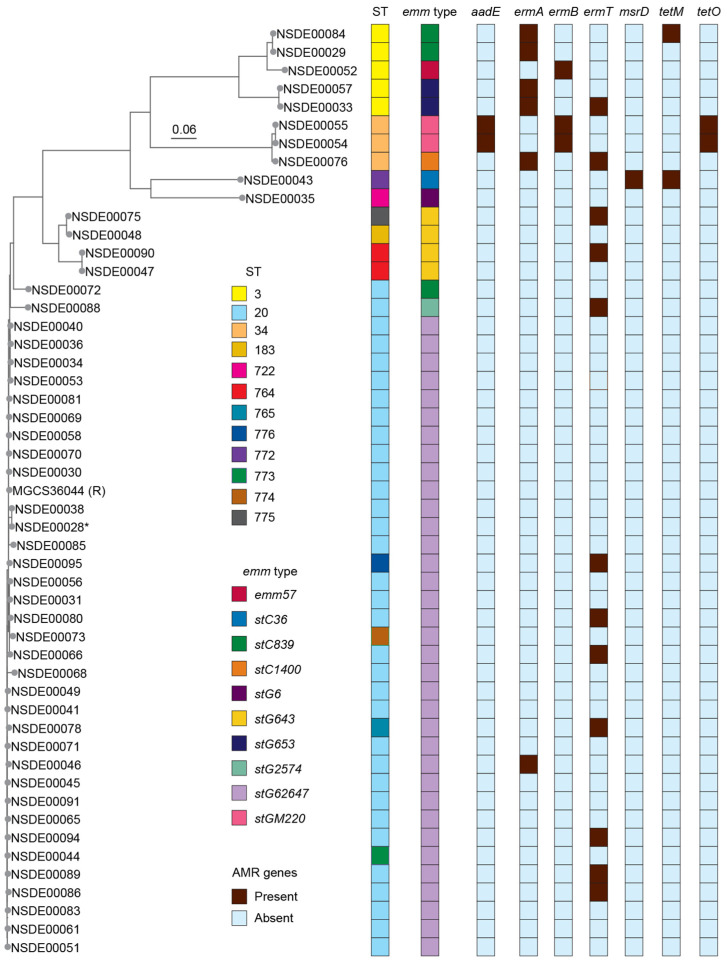
Phylogenetic relationships, MLST and *emm* type associations, and AMR gene content among *Streptococcus dysgalactiae* subsp. *equisimilis* isolates. The maximum-likelihood tree was constructed from 27,692 nonredundant core-genome SNPs identified relative to the reference strain MGCS36044 (ST20, *emm* type *stG62647*), indicated as “R.” The tree includes 49 invasive isolates recovered in Toronto (2018–2022) and one isolate from August 2017 (asterisk). Right-side panels indicate the corresponding STs, *emm* types, and presence of AMR genes.

To resolve genomic relationships beyond MLST and *emm* typing and to assess whether SDSE lineages circulating in Toronto exhibit internal structure or evidence of recombination, we next performed a core-genome-SNP-based phylogenetic analysis. SNPs were identified relative to the genome sequence of strain MGCS36044, an ST20 *stG62647* strain recovered in France from a human infection [10]. A reduced core-genome dataset of 27,692 nonredundant SNPs was used to construct a maximum likelihood phylogenetic tree providing high-resolution insights into the genetic diversity of the isolates (Figure 2).

Novel sequence types 776, 774, 765, and 773 clustered tightly with ST20 organisms, and they all belonged to *emm* type *stG62647*. This group exhibited a relatively modest level of genetic variability, suggesting clonal expansion. The median genetic distance separating the isolates was 308 SNPs (range: 27 to 1709 SNPs). All novel STs included in CC20 were genetically closer to the reference strain and the bulk of the ST20 isolates than two ST20 isolates belonging to *emm* types *stC839* and *stG2574* (Figure 2). These latter two isolates had the largest variances among the cluster with 1589 and 1709 SNPs respectively, suggesting unique evolutionary paths within this apparently homogenous group. Genetic diversity was more pronounced among ST3 SDSE isolates, which formed two relatively distant genomic subclades, with isolates belonging to three different *emm* types (Figure 2). The genetic distance among isolates in these subclades was, on average, 13,968 SNPs. ST34 organisms differed, on average, by 13,294 SNPs, and belonged to two different *emm* type (Figure 2). Four isolates formed two genomic subclades that included three different STs (ST183 and novel ST776 and ST775) but all isolates were of *emm* type *stG643* (Figure 2), suggesting potential horizontal *emm* transfer.

For comparative purposes, we next expanded our investigation to include genome sequences of 57 additional SDSE isolates from diverse geographic regions including from Manitoba, Canada, as well as Australia, China, France, Japan, Norway, Spain, the United Kingdom and the United States (Table S2). We identified 55,842 nonredundant core-genome SNPs across the extended dataset. Examination of the resulting phylogenies (Figure S2) revealed that Lancefield types G and A SDSE isolates were phylogenetically distinct from those in our collection. ST20 *stG62647* isolates from Toronto and other geographic areas were closely related, consistent with global clonal expansion of this genotype. Non-ST20, non-*stG62647* isolates from Toronto either clustered closely with strains from Manitoba (e.g., ST3 isolates), or were unique to the area (e.g., ST34 isolates and singletons), except for isolates belonging to ST183 of *emm* type *stG643*, and close derivatives, that clustered with an ST646 Australian strain of the same *emm* type (Figure S2).

### 3.5. ST20-stG62647 SDSE Isolates Are Associated with Older Patient Age, Whereas Sex and Infection Source Does Not Differ by SDSE Lineage

**Figure 3:**
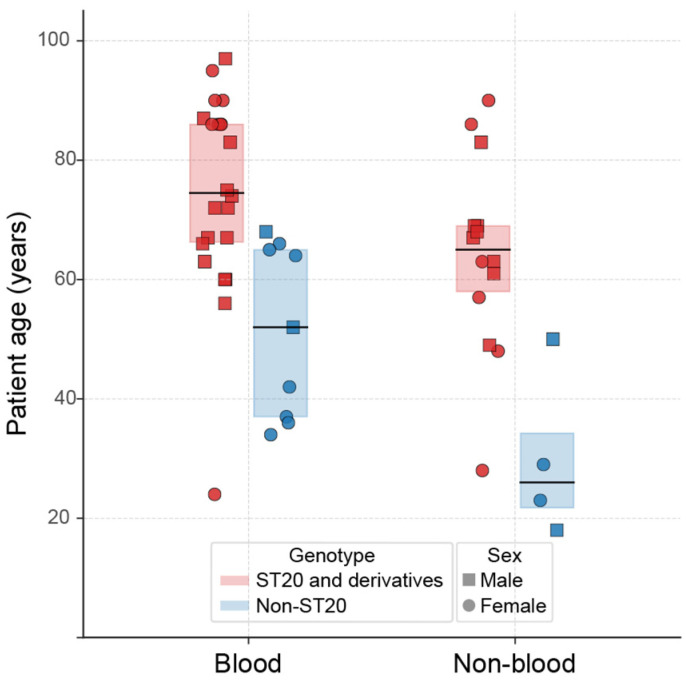
Distribution of *Streptococcus dysgalactiae* subsp. *equisimilis* genotypes by patient age and infection source. Each symbol represents one invasive case, grouped by isolate genotype (ST20 and its derivatives, red; non-ST20 lineages, blue) and type of infection (blood or non-blood). Circles denote female patients and squares denote male patients. Boxes represent the interquartile range (IQR) with the median shown as a horizontal line for each genotype. Patients infected with ST20 isolates were significantly older than those infected with non-ST20 isolates (overall *p* = 0.00023; blood *p* = 0.0023; non-blood *p* = 0.0125), whereas no significant differences were observed between blood and non-blood infections or between sexes.

To assess whether genomic lineages differed in their clinical or demographic distribution, we compared patient age, sex, and infection source across ST20 and non-ST20 lineages using the 49 cases with available genome data. ST20 and its closely related derivatives (ST765, ST773, ST774, and ST776) were identified in 22 of 31 (71%) blood isolates and 14 of 18 (78%) non-blood isolates, whereas non-ST20 genotypes were detected in nine and four isolates, respectively. When patient age was examined irrespective of infection source, ST20-lineage isolates were significantly associated with older patients compared with non-ST20 isolates (median 69 vs. 42 years; *p* = 0.0002) (Figure 3). We observed the same association in patients with bloodstream infections (median 74.5 vs. 52 years; *p* = 0.0023) and in those with non-blood infections (median 65 vs. 26 years; *p* = 0.0125).

To assess whether the observed association between ST20 lineage and patient age might be confounded by differences in the populations served by the two hospitals, we first compared the overall age distribution of sequenced SDSE cases between sites and found no significant difference (median 63 vs. 67 years; *p* = 0.28). We then repeated the analysis within each hospital. In both institutions, ST20-lineage isolates were associated with older patient age compared with non-ST20 isolates (hospital 1: median 67 vs. 36.5 years, *p* = 0.038; hospital 2: median 69 vs. 58 years, *p* = 0.013). Although the magnitude of the difference varied between sites, the direction of the association remained consistent across hospitals. Hospital identifiers for each case are provided in Table S1.

Defining genotypes by *emm* type rather than MLST produced similar results: *emm stG62647* isolates were recovered from significantly older patients than other *emm* types, both overall (median 69 vs. 50 years; *p* = 0.00047) and within blood (*p* = 0.0043) and non-blood (*p* = 0.018) infections (Figure S3). No statistically significant associations between patient sex and isolate genotype were observed. Together, these findings show that ST20, and, by extension, *stG62647* isolates were recovered from significantly older patients than non-ST20 isolates across both bloodstream and non-blood infections, whereas sex distribution and infection source did not differ by lineage.

### 3.6. SDSE Remains β-Lactam-Susceptible but Diverse Genes Harbored in Mobile Genetic Elements (MGEs) Mediate Macrolide and Tetracycline Resistance

To delineate the antimicrobial susceptibility profile of invasive SDSE in Toronto and identify the genetic determinants associated with resistance, we integrated phenotypic susceptibility testing with analyses of the distribution and genomic context of AMR genes. Susceptibility profiles were determined using Clinical and Laboratory Standards Institute (CLSI) disk-diffusion criteria, and resistance genes and mobile genetic elements (MGEs) were identified by screening genome assemblies for known AMR loci and associated integrative and conjugative elements (ICEs) or plasmid structures. All SDSE isolates were susceptible to penicillin, vancomycin, and gentamicin (Table 2). For tetracycline, 45 isolates (92%) were susceptible, one (2%) showed intermediate susceptibility, and three (6%) were resistant. The resistance gene *tetO* was identified in two of the resistant isolates, whereas *tetM* was detected in one intermediate and one susceptible isolate. Ten isolates (20%) were resistant to erythromycin and nine (18%) to clindamycin; four of the latter displayed inducible resistance in the D-test (Table 2 and Table S5). Resistant isolates carried macrolide-lincosamide-streptogramin B (MLS_B_) determinants, most commonly *ermT* (*n* = 12), *ermA* (*n* = 6), *ermB* (*n* = 2), and *msrD* (*n* = 1). However, the presence of *ermT* alone was not associated with erythromycin resistance and did not confer inducible clindamycin resistance as assessed by the D-test.

In the single *msrD*-positive isolate, the gene was chromosomally encoded whereas in all *ermT*-positive SDSE isolates, the gene was located on pGB2001-like plasmids [36]. *Tn*5252-family ICEs carried combinations of *aadE*, *tetO*, and *ermB*, whereas *tetM* was embedded within a *Tn*916-family ICE. A *Tn*1549-type ICE carried gene *ermB* in the remaining isolate with this gene (Figure S4). In five of six *ermA*-positive isolates, the gene was located within variable-length remnants of *Tn*1549-type ICEs, whereas in the remaining isolate *ermA* was found on a short contig compatible with an ICE remnant ; resolution of these elements would require long-read sequencing.

## 4. Discussion

The number of SDSE infections has increased worldwide [2,6,11,16,37]. Here, we characterized 56 cases of invasive SDSE infection identified in Toronto, Canada, between January 2018 and March 2022. Two-thirds of affected patients were over 60 years of age, consistent with population-based studies showing that invasive SDSE disease primarily affects older adults [12]. This age distribution may reflect, in part, the higher burden of comorbidities and the increased frequency of bloodstream infections in older individuals; however, because detailed clinical data were not available to this investigation, these factors could not be evaluated in the present study.

In our cohort, isolates of the ST20 (and closely related ST derivatives) *emm* type *stG62647* lineage predominated across all demographic groups, accounting for approximately three-quarters of sequenced isolates. This mirrors reports from multiple continents documenting the emergence of this lineage as a major cause of invasive SDSE infection, often linked to severe clinical manifestations [3,6,9,10,11]. We observed an association between genotype and patient age, with ST20 *stG62647* isolates being recovered more often from older patients, both overall and within blood and non-blood infections. This association remained when analyses were stratified by hospital, suggesting that it is unlikely to be explained solely by differences in the populations served by the two institutions. However, this finding should be interpreted with caution, as the number of non-ST20 cases was limited, reducing statistical power and making subgroup estimates sensitive to small sample sizes. The study design also did not allow adjustment for potential confounders such as comorbidities or healthcare exposure, and multiple subgroup comparisons were performed without correction, increasing the possibility of type I error. Therefore, while the observed association is consistent within the present dataset and with previous reports, our data do not allow us to determine whether it reflects biological differences between lineages or underlying patient-level factors. A previous study from Germany reported a sex-specific effect, with female patients infected with non-*stG62647* strains being on average 20 years younger than those infected with *stG62647* [11]. In our cohort, such sex-related differences were not observed, and the age association appeared consistent across both sexes.

Infections caused by non-ST20 lineages occurred in younger patients, but the underlying reasons remain unclear. Because detailed clinical outcome and comorbidity data were not available, we could not determine whether this pattern reflects differences in comorbidity profiles, disease severity, healthcare exposure, or other host- or setting-related factors, and its clinical significance therefore remains uncertain. In addition, a small proportion of cases (7/56, 12.5%) lacked viable isolates for sequencing. Comparison of available demographic and clinical variables for these cases did not reveal a consistent pattern suggesting a distinct subgroup or systematic bias affecting lineage prevalence estimates, although the small number of such cases limits definitive assessment.

In our collection, the non-ST20 lineages formed several well-defined, internally homogeneous clusters that together accounted for roughly one-quarter of sequenced isolates. Core-genome phylogeny showed that these lineages were genetically distinct from each other but displayed a relatively limited within-cluster diversity, consistent with a stable but less abundant set of co-circulating clonal groups accompanying the dominant ST20 *stG62647* lineage. This pattern contrasts with earlier Canadian data from Manitoba (2012–2014), which reported broader SDSE heterogeneity [16,17], and aligns with more recent international reports in which ST20 *stG62647* has expanded while non-ST20 lineages persist at a lower frequency [6,9,11]. The predominance of ST20 *stG62647* among SDSE circulating in Toronto may thus reflect a relatively recent epidemiologic shift, paralleling European and Asian trends. Retrospective studies will be needed to test this hypothesis. Together, our findings provide updated insights into the population structure of invasive SDSE in Canada and position the Toronto dataset within the broader North American and global epidemiological context. However, the composition of the comparative dataset is constrained by the availability of publicly deposited genomes and may therefore reflect uneven geographic sampling and overrepresentation of certain lineages, which should be considered when interpreting the broader phylogenetic context.

Overall, our isolates displayed a virulence gene repertoire similar to that reported in other regions, with no evidence of newly acquired virulence determinants [6,9,38]. All ST20 *stG62647* isolates carried an IS1548 insertion disrupting *silB*, a regulatory gene within the *sil* locus, while all but one non-ST20 isolates had an intact *silB* gene. Interestingly, the serine protease gene *scpB* was detected only among isolates with an intact *silB*, further supporting the genomic distinction between *silB*-disrupted and *silB*-intact lineages. Apart from these features, the virulence profile of ST20 and non-ST20 lineages was broadly similar. Thus, our findings are consistent with previous studies [6,9,38] suggesting that regulatory differences associated with *silB* disruption may contribute to the biological distinctiveness of the ST20 *stG62647* lineage. However, our data do not allow us to determine whether this feature is associated with transmission, disease severity, or other clinically relevant outcomes. Because detailed patient clinical metadata were not available, the clinical significance of *silB* disruption and other lineage-associated genomic traits remains uncharacterized in the present study.

All isolates in our collection were susceptible to penicillin and vancomycin, providing continued reassurance regarding first-line therapy. However, our findings contrast with reports from Denmark and Finland describing isolates with reduced penicillin susceptibility [37,39]. Resistance to tetracyclines, macrolides, and lincosamides among Toronto isolates paralleled recent surveys from Japan, Finland, and Norway [2,14], highlighting an expanding resistome within SDSE populations. Genomic analysis revealed multiple MGEs carrying AMR genes among our isolates.

The *ermT* gene was consistently carried on pGB2001-like plasmids, but in our collection it was not associated with erythromycin resistance and did not confer inducible clindamycin resistance. This genotype–phenotype discordance is notable and likely reflects variation in *ermT* expression. Expression of *erm* genes is typically regulated through a translational attenuation mechanism involving an upstream leader peptide and alternative mRNA secondary structures that control access to the ribosome-binding site, as demonstrated for *ermT* [40]. In the absence of macrolides, these structures prevent translation, whereas antibiotic-induced ribosome stalling promotes a conformational switch that enables erm expression [40]. Variations in the regulatory region, including mutations, deletions, or rearrangements, can shift this system toward inducible or constitutive expression states or impair expression altogether, thereby modulating resistance phenotypes [40,41]. In addition, experimental work has shown that *ermT*-carrying plasmids can confer macrolide and lincosamide resistance in heterologous hosts, indicating that genetic context and regulatory architecture are critical determinants of phenotypic expression [40,41]. The absence of a resistance phenotype in our isolates despite the presence of *ermT* therefore suggests that the gene may be transcriptionally silent, poorly expressed, or conditionally inducible under conditions not captured by routine susceptibility testing. However, the present study was not designed to investigate the regulatory or functional basis of *ermT* expression, and resolving this question would require dedicated mechanistic analyses beyond the scope of this work. 

Genes *tetM*, *tetO*, *ermA*, *ermB*, and *aadE* were integrated within different ICE families. These different MGE-borne resistance modules underscore the versatility of SDSE as both a reservoir and conduit for AMR genes within the genus *Streptococcus* [9,10], including the potential for horizontal transfer of these determinants to other streptococcal species in shared ecological niches. Because genome assemblies were generated from short-read sequencing data, the complete structure of mobile genetic elements could not be fully resolved, and associations between resistance genes and specific genetic platforms should therefore be interpreted with caution.

Taken together, our findings show that invasive SDSE infections in Toronto are dominated by the globally expanding ST20 *stG62647* lineage, with other lineages persisting at a lower frequency and being recovered more often from younger patients in this dataset. The consistent *silB* disruption among ST20 isolates further supports the genomic distinction of this lineage, although the clinical implications of this and other lineage-associated features could not be assessed in the absence of detailed patient-level data. Although β-lactams remain fully active, the diversity of ICE-associated AMR genes highlights the potential for rapid resistance shifts under antimicrobial pressure. Continued genomic and clinical surveillance will be essential to track SDSE evolution, evaluate emerging resistance mechanisms, and guide public health responses.

## Figures and Tables

**Table 1:** Demographic characteristics of patients and sources of invasive SDSE isolates **.**

Patient Characteristics	Value (%)
Total	56 (100)
Mean age	60.8 ± 2.0 (0–97) *
Median age	65
Age groups	
<18	1 * (2)
18–59	19 (34)
60–74	22 (39)
≥75	14 (25)
Sex	
M	29 (52)
F	27 (48)
Isolates recovered from	
Abscess	11 (20)
Ascites	2 (4)
Blood	35 (62)
Operating room swab or tissue	8 (14)

* Includes one infection in a newborn (<28 days).

**Table 2:** Antimicrobial susceptibility of SDSE isolates.

Antibiotic	Breakpoints			Number of Isolates			AMR Genes
	Sensitive	Intermediate	Resistant	Sensitive	Intermediate	Resistant	
Penicillin G	≥24 mm	21–23 mm	≤20 mm	49	0	0	
Vancomycin	≥17 mm	-	-	49	0	0	
Gentamicin ^a^	15	13–14	12	49	0	0	*aadE* (*n* = 2)
Tetracycline	≥23 mm	19–22 mm	≤18 mm	45	1	3	*tetM* (*n* = 2); *tetO* (*n* = 2)
Clindamycin	≥19 mm	16–18 mm	≤15 mm	40 ^b^	0	9	*ermT* (*n* = 12); *ermA* (*n* = 6); *ermB* (*n* = 2); *msrD* (*n* = 1)
Erythromycin	≥ 21 mm	16–20 mm	≤15 mm	39	0	10	*ermT* (*n* = 12); *ermA* (*n* = 6); *ermB* (*n* = 2); *msrD* (*n* = 1)

^a^ Gentamicin disk diffusion breakpoints are not defined for β-hemolytic streptococci by CLSI. Values for *Staphylococcus aureus* were used for comparative purposes only. ^b^ Four isolates were susceptible by Kirby–Bauer testing but showed inducible clindamycin resistance in the D-test after erythromycin induction.

## Data Availability

Sequencing data have been submitted to NCBI under BioProject accession number PRJNA1099199. Table S1 lists the individual accession numbers for NCBI’s Sequence Read Archive.

## Supplementary Materials

The following supporting information can be downloaded at: https://www.mdpi.com/article/10.3390/microorganisms14040878/s1, Figure S1: Epidemiologic distribution of invasive *Streptococcus dysgalactiae* subsp. *equisimilis* (SDSE) infections in Toronto, Ontario, Canada, 2018–2022; Figure S2: Inferred genetic relationships among SDSE isolates from Toronto and global regions; Figure S3: Distribution of *emm* genotypes by patient age and infection source; Figure S4: Distribution of antimicrobial resistance (AMR) genes within integrative conjugative elements (ICEs) across SDSE isolates; Table S1: SDSE isolates used in this study; Table S2: Additional SDSE genomes included in this study; Table S3: Multilocus sequence typing (MLST) sequence types identified among the SDSE isolates used in this study; Table S4: Superantigens and selected virulence factors identified among the SDSE isolates used in this study; Table S5: Kirby–Bauer disk-diffusion test results and antimicrobial resistance genes detected among SDSE isolates.

## Abbreviations

The following abbreviations are used in this manuscript:

AMR	Antimicrobial resistance
BLAST	Basic Local Alignment Search Tool
CARD	Comprehensive Antibiotic Resistance Database
CC	Clonal complex
CLSI	Clinical and Laboratory Standards Institute
DNA	Deoxyribonucleic acid
ICE	Integrative and conjugative element
IQR	Interquartile range
MGE	Mobile genetic element
MLSB	Macrolide–lincosamide–streptogramin B
MLST	Multilocus sequence typing
PCR	Polymerase chain reaction
REB	Research Ethics Board
SDSE	*Streptococcus dysgalactiae* subsp. *equisimilis*
SH	Shimodaira–Hasegawa
SNP	Single nucleotide polymorphism
ST	Sequence type
TE	Tris–EDTA buffer
WGS	Whole-genome sequencing
