# Supplementary material for: Dominance of the ST20 stG62647 Lineage Among Invasive Streptococcus dysgalactiae subsp. equisimilis Infections in Toronto, Canada
Source: Microorganisms. 2026 Apr 14;14(4):878. doi: 10.3390/microorganisms14040878 (PMC13119170; doi:10.3390/microorganisms14040878)
Supplement: Supplementary file 1 [file microorganisms-14-00878-s001.zip › Figure-S1.pdf]

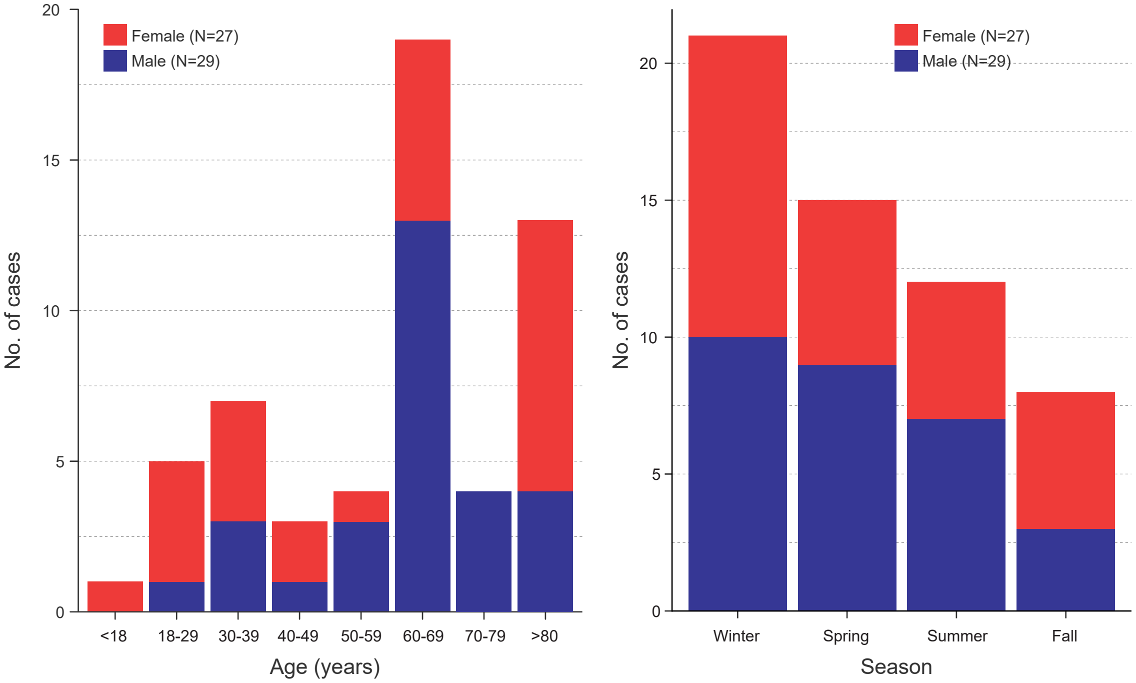

Figure S1. Epidemiologic distribution of invasive SDSE infections in Toronto, Ontario, Canada, 2018–2022**.** A) Age and sex distribution of patients with invasive SDSE infections. Bars show the number of cases in 10-year age intervals, separated by sex (female = 27, male = 29). Most infections occurred in adults ≥ 60 years of age, and age distributions were similar between sexes. B) Seasonal distribution of invasive SDSE cases. Bars show the number of infections by season, separated by sex. Seasons were defined as winter (December–February), spring (March–May), summer (June–August), and fall (September–November). Although seasonal differences were not statistically significant, more cases occurred in winter and spring, suggesting a mild seasonal trend.
