# Supplementary material for: Dominance of the ST20 stG62647 Lineage Among Invasive Streptococcus dysgalactiae subsp. equisimilis Infections in Toronto, Canada
Source: Microorganisms. 2026 Apr 14;14(4):878. doi: 10.3390/microorganisms14040878 (PMC13119170; doi:10.3390/microorganisms14040878)
Supplement: Supplementary file 1 [file microorganisms-14-00878-s001.zip › Figure-S3.pdf]

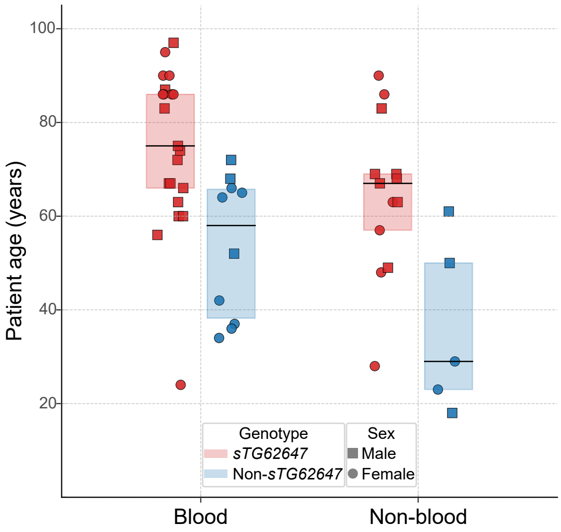

Figure S3. Distribution of *emm* genotypes by patient age and infection source. Each symbol represents one invasive SDSE case, grouped by *emm* genotype (*stG62647*, red; other emm types, blue) and type of infection (blood or non-blood). Squares denote male patients and circles denote female patients. Boxes represent the interquartile range (IQR) with the median shown as a horizontal line for each *emm* genotype. Patients infected with *stG62647* isolates were significantly older than those infected with other *emm* types (overall p = 0.00047; blood p = 0.0043; non-blood p = 0.018), whereas no significant differences were observed between blood and non-blood infections or between sexes.
