# Supplementary material for: Dominance of the ST20 stG62647 Lineage Among Invasive Streptococcus dysgalactiae subsp. equisimilis Infections in Toronto, Canada
Source: Microorganisms. 2026 Apr 14;14(4):878. doi: 10.3390/microorganisms14040878 (PMC13119170; doi:10.3390/microorganisms14040878)
Supplement: Supplementary file 1 [file microorganisms-14-00878-s001.zip › Figure-S4.pdf]

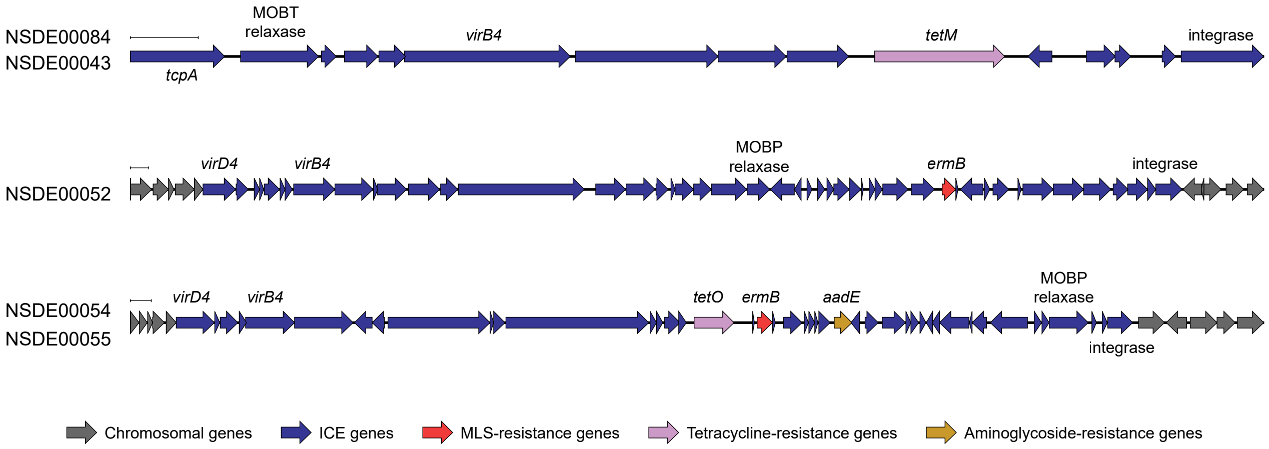

Figure S4. Distribution of antimicrobial resistance (AMR) genes within integrative conjugative elements (ICEs) across different SDSE isolates used in this study. Illustrated is the assortment of AMR genes harbored by different ICEs in several SDSE isolates. The AMR genes depicted include *tetM* and *tetO*, which confer resistance to tetracycline, *ermB*, associated with resistance to macrolide-lincosamide-streptogramin (MLS) antibiotics; and *aadE*, providing resistance against specific aminoglycosides. Key ICE genes, such as integrases and relaxases from the mobilizable conjugative transposon (MOBT) and mobilizable plasmid (MOBP) families that promote the mobility of these elements, are depicted for reference. The ICEs identified belong to various groups associated with well-known transposons: *Tn*916 (top) found in isolates NSDE00084 and NSDE00043; *Tn*1549 (middle), in isolate NSDE00052, and *Tn*5252 (bottom), in isolates NSDE00054 and NSDE00055. The bars indicate 1,000 bp.
