# Supplementary material for: Dominance of the ST20 stG62647 Lineage Among Invasive Streptococcus dysgalactiae subsp. equisimilis Infections in Toronto, Canada
Source: Microorganisms. 2026 Apr 14;14(4):878. doi: 10.3390/microorganisms14040878 (PMC13119170; doi:10.3390/microorganisms14040878)
Supplement: Supplementary file 1 [file microorganisms-14-00878-s001.zip › Table_S1.pdf]

Table S1. SDSE isolates used in this study.

Isolate	Hospital	Source of isolation	Date of isolation (MM/YYYY)	Patient Sex	Patient Age	Whole-genome sequencing data				Kraken taxonomic identification		MLST Sequence Type	*emm* typing
						Number of reads	Calculated average depth of coverage[a]	Genome size (bp)[b]	SRA accession number[c]	% of reads matching *S. dysgalactiae* group	% of reads matching *S. dysgalactiae* subsp. *equisimilis*		
NSDE00029	2	Blood	01/2018	F	64	4182908	277	2165301	SRR28625598	50.36	20.07	3	*stC839*
NSDE00030	2	Abscess	01/2018	F	48	4025128	266	2143613	SRR28625587	49.85	19.39	20	*stG62647*
NSDE00031	2	Abscess	03/2018	M	69	3609956	243	2140479	SRR28625576	49.09	18.5	20	*stG62647*
NSDE00033	2	Blood	03/2018	M	52	4397754	270	2216508	SRR28625565	45.74	17.94	3	*stG653*
NSDE00034	2	Blood	03/2018	M	97	4639382	319	2100870	SRR28625554	50.34	18.99	20	*stG62647*
NSDE00035	2	Abscess	04/2018	M	18	4650370	296	2231153	SRR28625553	47.02	15.6	722	*stG6*
NSDE00036	2	Blood	04/2018	F	24	3689216	246	2144864	SRR28625552	49.93	19.26	20	*stG62647*
NSDE00038	2	Blood	04/2018	M	72	4440360	303	2142339	SRR28625551	49.41	18.52	20	*stG62647*
NSDE00040	2	Blood	07/2018	F	90	4738832	314	2183960	SRR28625550	48.92	18.47	20	*stG62647*
NSDE00041	2	Blood	09/2018	M	87	3692238	248	2150055	SRR28625597	50.23	18.41	20	*stG62647*
NSDE00043	1	Blood	10/2018	F	42	3654520	236	2190422	SRR28625596	45.91	15.27	772	*stC36*
NSDE00044	2	Blood	10/2018	M	74	4303732	290	2140457	SRR28625595	51.09	19.02	773	*stG62647*
NSDE00045	1	OR[d] Swab/Tissue	12/2018	M	63	4219974	291	2104035	SRR28625594	50.29	18.98	20	*stG62647*
NSDE00046	2	Abscess	12/2018	F	57	4052992	278	2107549	SRR28625593	49.61	18.59	20	*stG62647*
NSDE00047	1	Blood	12/2018	F	34	5199974	340	2218269	SRR28625592	49.63	19.13	764	*stG643*
NSDE00048	2	Blood	12/2018	F	66	5130044	346	2147323	SRR28625591	52.66	20.87	183	*stG643*
NSDE00049	1	Abscess	12/2018	F	63	5083828	325	2272718	SRR28625590	47.9	18.07	20	*stG62647*
NSDE00051	2	Blood	02/2019	M	56	4957646	323	2220356	SRR28625589	50.07	18.76	20	*stG62647*
NSDE00052	2	Blood	02/2019	M	68	4520702	311	2075838	SRR28625588	51.01	21.09	3	*emm57*
NSDE00053	2	Abscess	03/2019	M	67	4961784	316	2219485	SRR28625586	49.41	19.9	20	*stG62647*
NSDE00054	1	Abscess	05/2019	F	29	4651024	320	2088690	SRR28625585	49.4	16.98	34	*stGM220*
NSDE00055	2	OR Swab/Tissue	07/2019	M	50	3791222	261	2090726	SRR28625584	49.43	17.01	34	*stGM220*
NSDE00056	2	Blood	07/2019	M	60	4399430	293	2167301	SRR28625583	48.41	18.17	20	*stG62647*
NSDE00057	2	Blood	08/2019	F	65	3252882	206	2274643	SRR28625582	47.1	18.21	3	*stG653*
NSDE00058	2	Blood	09/2019	M	63	4441104	280	2231015	SRR28625581	49.19	18.56	20	*stG62647*
NSDE00061	2	Blood	11/2019	F	90	4358210	280	2272270	SRR28625580	48.05	18.31	20	*stG62647*
NSDE00065	1	OR Swab/Tissue	02/2020	M	49	4269738	293	2131773	SRR28625579	49.87	18.74	20	*stG62647*

Isolate	Hospital	Source of isolation	Date of isolation (MM/YYYY)	Patient Sex	Patient Age	Whole-genome sequencing data				Kraken taxonomic identification		MLST Sequence Type	*emm* typing
						Number of reads	Calculated average depth of coverage[a]	Genome size (bp)[b]	SRA accession number[c]	% of reads matching *S. dysgalactiae* group	% of reads matching *S. dysgalactiae* subsp. *equisimilis*		
NSDE00066	2	Ascites	02/2020	M	69	4922282	337	2139681	SRR28625578	49.95	18.8	20	*stG62647*
NSDE00068	1	Blood	03/2020	F	95	3997198	273	2106300	SRR28625577	49.92	18.81	20	*stG62647*
NSDE00069	2	Blood	04/2020	M	83	4483272	308	2104302	SRR28625575	49.8	18.8	20	*stG62647*
NSDE00070	2	Blood	05/2020	F	86	4100874	256	2273297	SRR28625574	50.29	19.95	20	*stG62647*
NSDE00071	1	Blood	07/2020	M	67	3243106	221	2136258	SRR28625573	50.94	20.08	20	*stG62647*
NSDE00072	1	Blood	07/2020	M	72	3290662	208	2192593	SRR28625572	50.06	19.45	20	*stC839*
NSDE00073	2	Blood	08/2020	M	67	4417168	296	2165202	SRR28625571	50.6	18.55	774	*stG62647*
NSDE00075	1	Ascites	08/2020	F	90	4038530	264	2248310	SRR28625570	50.03	20.38	775	*stG643*
NSDE00076	1	Blood	10/2020	F	37	2840414	195	2115565	SRR28625569	48.74	16.24	34	*stC1400*
NSDE00078	2	Blood	12/2020	F	86	3609612	237	2190548	SRR28625568	50.45	20.35	765	*stG62647*
NSDE00080	2	OR Swab/Tissue	01/2021	M	68	3540922	244	2105502	SRR28625567	50.33	18.97	20	*stG62647*
NSDE00081	2	Abscess	01/2021	F	86	4136562	268	2194973	SRR28625566	49.91	18.77	20	*stG62647*
NSDE00083	2	OR Swab/Tissue	01/2021	M	83	4014958	277	2138559	SRR28625564	50.17	18.92	20	*stG62647*
NSDE00084	2	Abscess	02/2021	F	23	4645428	306	2170120	SRR28625563	49.23	19.39	3	*stC839*
NSDE00085	2	Blood	03/2021	M	66	5753828	385	2151472	SRR28625562	49.95	18.88	20	*stG62647*
NSDE00086	2	Abscess	05/2021	F	28	4394890	294	2156219	SRR28625561	49.74	19.36	20	*stG62647*
NSDE00088	2	OR Swab/Tissue	07/2021	M	61	4187990	288	2114096	SRR28625560	49.66	18.65	20	*stG2574*
NSDE00089	1	Blood	07/2021	M	75	3990290	274	2115798	SRR28625559	49.79	18.73	20	*stG62647*
NSDE00090	1	Blood	07/2021	F	36	4424356	294	2189428	SRR28625558	49.95	19.2	764	*stG643*
NSDE00091	2	Blood	11/2021	F	86	3966662	268	2155803	SRR28625557	50.68	18.68	20	*stG62647*
NSDE00094	2	Blood	01/2022	M	60	4094942	281	2112402	SRR28625556	50.11	18.86	20	*stG62647*
NSDE00095	2	Blood	03/2022	F	86	4102718	282	2138809	SRR28625555	50.66	19.16	776	*stG62647*
NSDE00060	2	Blood	10/2019	F	65	N/A	N/A	N/A	N/A	N/A	N/A	N/A	N/A
NSDE00062	2	Blood	12/2019	M	33	N/A	N/A	N/A	N/A	N/A	N/A	N/A	N/A
NSDE00063	2	Abscess	02/2020	M	39	N/A	N/A	N/A	N/A	N/A	N/A	N/A	N/A
NSDE00064	1	Blood	02/2020	F	0	N/A	N/A	N/A	N/A	N/A	N/A	N/A	N/A
NSDE00074	2	OR Swab/Tissue	08/2020	F	34	N/A	N/A	N/A	N/A	N/A	N/A	N/A	N/A
NSDE00077	2	OR Swab/Tissue	12/2020	F	68	N/A	N/A	N/A	N/A	N/A	N/A	N/A	N/A

Isolate	Hospital	Source of isolation	Date of isolation (MM/YYYY)	Patient Sex	Patient Age	Whole-genome sequencing data				Kraken taxonomic identification		MLST Sequence Type	*emm* typing
						Number of reads	Calculated average depth of coverage[a]	Genome size (bp)[b]	SRA accession number[c]	% of reads matching *S. dysgalactiae* group	% of reads matching *S. dysgalactiae* subsp. *equisimilis*		
NSDE00087	2	Blood	05/2021	M	38	N/A	N/A	N/A	N/A	N/A	N/A	N/A	N/A

[a]Calculated based on an average genome size for SDSE of 2,1 Mbp.

[b]Sum of all *de novo* assembled contigs for each isolate.

[c]National Center for Biotechnology Information Sequence Read Archive (https://www.ncbi.nlm.nih.gov/sra/) accession number.

[d]OR: Operating room.
