# Supplementary material for: Dominance of the ST20 stG62647 Lineage Among Invasive Streptococcus dysgalactiae subsp. equisimilis Infections in Toronto, Canada
Source: Microorganisms. 2026 Apr 14;14(4):878. doi: 10.3390/microorganisms14040878 (PMC13119170; doi:10.3390/microorganisms14040878)
Supplement: Supplementary file 1 [file microorganisms-14-00878-s001.zip › Table_S2.pdf]

Table S2. Additional SDSE genomes included in this study.

Strain name	SRA accession number[a]	Country	Lancefield antigen	*emm* type	ST[b]
NSDE00028	SRR28625599	Canada	C	*stG62647*	20
4	SRX1851293	Canada	C	*stG62647*	20
5	SRX1851304	Canada	C	*stG62647*	20
6	SRX1851315	Canada	C	*stC839*	3
18	SRX1851279	Canada	C	*stG62647*	20
24	SRX1851284	Canada	C	*stG62647*	20
27	SRX1851287	Canada	G	*stG245*	282
28	SRX1851288	Canada	G	*stG166b*	25
29	SRX1851289	Canada	C	*stG62647*	20
31	SRX1851291	Canada	G	*stG6*	12
32	SRX1851292	Canada	G	*stG653*	448
33	SRX1851294	Canada	C	*stG6*	275
35	SRX1851296	Canada	G	*stG480*	8
36	SRX1851297	Canada	G	*stG480*	282
37	SRX1851298	Canada	C	*stG62647*	20
38	SRX1851299	Canada	C	*stG62647*	20
39	SRX1851300	Canada	G	*stG6*	52
48	SRX1851310	Canada	G	*stG6*	154
50	SRX1851312	Canada	G	*stG245*	17
52	SRX1851314	Canada	G	*stC74a*	29
53	SRX1851316	Canada	C	*stG62647*	20
54	SRX1851317	Canada	C	*stG62647*	20
55	SRX1851318	Canada	C	*stG62647*	20
59	SRX1851322	Canada	G	*stGPL1*	323
60	SRX1851323	Canada	C	*stG62647*	20
61	SRX1851324	Canada	G	*stG10*	15
64	SRX1851328	Canada	C	*stG62647*	20
20162140	SRX3023016	United States	A	*stG485*	128
20163069	SRX3022995	United States	A	*stG652*	128
AKSDE4288	SRX1984677	United States	C	*stG485*	69
GGS124	ERX105038	United Kingdom	G	*stG480*	67
iSDSE_NORM105	ERX10147175	Norway	C	*stG62647*	20

Strain name	SRA accession number[a]	Country	Lancefield antigen	*emm* type	ST[b]
iSDSE_NORM107	ERX10147180	Norway	G	*stC74a*	77
iSDSE_NORM110	ERX10147188	Norway	C	*stG6*	20
iSDSE_NORM112	ERX10147193	Norway	G	*stG485*	596
iSDSE_NORM117	ERX10147196	Norway	G	*stC345*	17
KNZ16	DRX445892	Japan	G	*stG166b*	148
KUSD150	DRX348603	Japan	G	*stG245*	25
KUSD152	DRX348605	Japan	G	*stC74a*	29
KUSD153	DRX348606	Japan	G	*stG6*	25
KUSD157	DRX348610	Japan	G	*stG485*	29
MGCS35957	SRX19098857	France	C	*stG62647*	20
MGCS36083	SRX19098860	France	C	*stG62647*	20
MGCS36089	SRX19098861	France	C	*stG62647*	20
MGGS36055	SRX19098859	France	G	*stC74a*	17
MMC234	SRX23975710	Australia	C	*stG643*	646
NS9587SE	ERX105052	United Kingdom	C	*stC7505*	10
SDSE-HGUGM-3081M	ERX9692730	Spain	G	*stG10*	15
SDSE-HUB-05387	ERX9692740	Spain	G	*stG166b*	31
SDSE-HUB-05597	ERX9692741	Spain	G	*stG245*	127
SDSE-HUB-30963	ERX9692747	Spain	G	*stG2574*	15
SDSE-HUD-70956031	ERX9692750	Spain	G	*stG6*	15
SE_G111	ERX105049	United Kingdom	G	*stC46*	134
SE_YarC5	ERX105039	United Kingdom	G	*stG10*	15
SE_YarG1	ERX105041	United Kingdom	C	*stC839*	3
UT_10236	SRX18208121	United States	C	*stG62647*	20
UT_120444	SRX18208120	United States	G	*stG245*	17
WCHSDSE-1	SRX1047123	China	G	*stG211*	44

[a]National Center for Biotechnology Information Sequence Read Archive (https://www.ncbi.nlm.nih.gov/sra/) accession number.
[b]ST: sequence type as determined by *in silico*-based multilocus sequence typing.
