# Supplementary material for: Dominance of the ST20 stG62647 Lineage Among Invasive Streptococcus dysgalactiae subsp. equisimilis Infections in Toronto, Canada
Source: Microorganisms. 2026 Apr 14;14(4):878. doi: 10.3390/microorganisms14040878 (PMC13119170; doi:10.3390/microorganisms14040878)
Supplement: Supplementary file 1 [file microorganisms-14-00878-s001.zip › Table_S3.pdf]

Table S3. Multilocus Sequence Typing Sequence Types identified among the SDSE isolates used in this study.

ST[a]	Gene allele number							Number of isolates
	gki	*gtr*	*murI*	*mutS*	*recP*	*xpt*	*atoB*	
3	5	3	4	1	6	2	1	5
20	3	3	2	8	9	6	6	31
34	3	7	4	1	14	15	10	3
183	3	3	2	8	9	8	2	1
722	4	2	4	1	83	1	2	1
764	19	3	2	8	9	8	6	2
765	3	3	2	8	9	1	6	1
772	85	3	34	1	45	60	1	1
773	3	3	2	8	9	6	68	1
774	3	3	2	8	9	143	6	1
775	3	3	2	8	90	8	2	1
776	3	3	2	8	9	144	6	1

[a]ST: sequence type as determined by *in silico*-based multilocus sequence typing.
