# Supplementary material for: Dominance of the ST20 stG62647 Lineage Among Invasive Streptococcus dysgalactiae subsp. equisimilis Infections in Toronto, Canada
Source: Microorganisms. 2026 Apr 14;14(4):878. doi: 10.3390/microorganisms14040878 (PMC13119170; doi:10.3390/microorganisms14040878)
Supplement: Supplementary file 1 [file microorganisms-14-00878-s001.zip › Table_S4.pdf]

Table S4. Superantigens and selected virulence factors identified among the SDSE isolates used in this study.

Isolate	Superantigen													Virulence factor						
	speA	*speC*	*speF*	*speG*	*speH*	*speI*	*speJ*	*speK*	*speL*	*speM*	*smez*	*ssa*	*sdm*	*hasC*	*ska*	*slo*	*scpA*	*scpB*	*sda*	*silB*
NSDE00029	-	-	-	+	-	-	-	-	-	-	-	-	-	+	+	+	-	+	-	+
NSDE00030	-	-	-	+	-	-	-	-	-	-	-	-	-	+	+	+	+	-	-	int[a]
NSDE00031	-	-	-	+	-	-	-	-	-	-	-	-	-	+	+	+	+	-	+	int
NSDE00033	-	-	-	-	-	-	-	-	-	-	-	-	-	+	+	+	-	+	+	+
NSDE00034	-	-	-	+	-	-	-	-	-	-	-	-	-	+	+	+	+	-	-	int
NSDE00035	-	-	-	-	-	-	-	-	-	-	-	-	-	+	+	+	+	-	-	+
NSDE00036	-	-	-	+	-	-	-	-	-	-	-	-	-	+	+	+	+	-	-	int
NSDE00038	-	-	-	+	-	-	-	-	-	-	-	-	-	+	+	+	+	-	-	int
NSDE00040	-	-	-	+	-	-	-	-	-	-	-	-	-	+	+	+	+	-	-	int
NSDE00041	-	-	-	+	-	-	-	-	-	-	-	-	-	+	+	+	+	-	-	int
NSDE00043	-	-	-	-	-	-	-	-	-	-	-	-	-	+	+	+	+	-	+	-[b]
NSDE00044	-	-	-	+	-	-	-	-	-	-	-	-	-	+	+	+	+	-	-	int
NSDE00045	-	-	-	+	-	-	-	-	-	-	-	-	-	+	+	+	+	-	-	int
NSDE00046	-	-	-	+	-	-	-	-	-	-	-	-	-	+	+	+	+	-	-	int
NSDE00047	-	-	-	+	-	-	-	-	-	-	-	-	-	+	+	+	+	-	-	+
NSDE00048	-	-	-	+	-	-	-	-	-	-	-	-	-	+	+	+	+	-	-	+
NSDE00049	-	-	-	+	-	-	-	-	-	-	-	-	-	+	+	+	+	-	-	int
NSDE00051	-	-	-	+	-	-	-	-	-	-	-	-	-	+	+	+	+	-	-	int
NSDE00052	-	-	-	+	-	-	-	-	-	-	-	-	-	+	+	+	-	+	-	+
NSDE00053	-	-	-	+	-	-	-	-	-	-	-	-	-	+	+	+	+	-	+	int
NSDE00054	-	-	-	-	-	-	-	-	-	-	-	-	-	+	+	+	+	-	-	+
NSDE00055	-	-	-	-	-	-	-	-	-	-	-	-	-	+	+	+	+	-	-	+
NSDE00056	-	-	-	+	-	-	-	-	-	-	-	-	-	+	+	+	+	-	-	int
NSDE00057	-	-	-	-	-	-	-	-	-	-	-	-	-	+	+	+	-	+	-	+
NSDE00058	-	-	-	+	-	-	-	-	-	-	-	-	-	+	+	+	+	-	-	int
NSDE00061	-	-	-	+	-	-	-	-	-	-	-	-	-	+	+	+	+	-	-	int
NSDE00065	-	-	-	+	-	-	-	-	-	-	-	-	-	+	+	+	+	-	-	int

Isolate	Superantigen													Virulence factor						
	speA	*speC*	*speF*	*speG*	*speH*	*speI*	*speJ*	*speK*	*speL*	*speM*	*smez*	*ssa*	*sdm*	*hasC*	*ska*	*slo*	*scpA*	*scpB*	*sda*	*silB*
NSDE00066	-	-	-	+	-	-	-	-	-	-	-	-	-	+	+	+	+	-	-	int
NSDE00068	-	-	-	+	-	-	-	-	-	-	-	-	-	+	+	+	+	-	-	int
NSDE00069	-	-	-	+	-	-	-	-	-	-	-	-	-	+	+	+	+	-	-	int
NSDE00070	-	-	-	+	-	-	-	-	-	-	-	-	-	+	+	+	+	-	-	int
NSDE00071	-	-	-	+	-	-	-	-	-	-	-	-	-	+	+	+	+	-	-	int
NSDE00072	-	-	-	+	-	-	-	-	-	-	-	-	-	+	+	+	+	-	-	int
NSDE00073	-	-	-	+	-	-	-	-	-	-	-	-	-	+	+	+	+	-	-	int
NSDE00075	-	-	-	+	-	-	-	-	-	-	-	-	-	+	+	+	+	-	-	+
NSDE00076	-	-	-	-	-	-	-	-	-	-	-	-	-	+	+	+	+	-	-	+
NSDE00078	-	-	-	+	-	-	-	-	-	-	-	-	-	+	+	+	+	-	-	int
NSDE00080	-	-	-	+	-	-	-	-	-	-	-	-	-	+	+	+	+	-	-	int
NSDE00081	-	-	-	+	-	-	-	-	-	-	-	-	-	+	+	+	+	-	-	int
NSDE00083	-	-	-	+	-	-	-	-	-	-	-	-	-	+	+	+	+	-	-	int
NSDE00084	-	-	-	+	-	-	-	-	-	-	-	-	-	+	+	+	-	+	-	+
NSDE00085	-	-	-	+	-	-	-	-	-	-	-	-	-	+	+	+	+	-	-	int
NSDE00086	-	-	-	+	-	-	-	-	-	-	-	-	-	+	+	+	+	-	-	int
NSDE00088	-	-	-	+	-	-	-	-	-	-	-	-	-	+	+	+	+	-	-	int
NSDE00089	-	-	-	+	-	-	-	-	-	-	-	-	-	+	+	+	+	-	-	int
NSDE00090	-	-	-	+	-	-	-	-	-	-	-	-	-	+	+	+	+	-	-	+
NSDE00091	-	-	-	+	-	-	-	-	-	-	-	-	-	+	+	+	+	-	-	int
NSDE00094	-	-	-	+	-	-	-	-	-	-	-	-	-	+	+	+	+	-	-	int
NSDE00095	-	-	-	+	-	-	-	-	-	-	-	-	-	+	+	+	+	-	-	int

[a]int indicates that the gene *silB* is interrupted likely from insertion of an *IS1548* element.

[b]None of the genes in the *sil* locus were detected in this isolate
