# Supplementary material for: Dominance of the ST20 stG62647 Lineage Among Invasive Streptococcus dysgalactiae subsp. equisimilis Infections in Toronto, Canada
Source: Microorganisms. 2026 Apr 14;14(4):878. doi: 10.3390/microorganisms14040878 (PMC13119170; doi:10.3390/microorganisms14040878)
Supplement: Supplementary file 1 [file microorganisms-14-00878-s001.zip › Table_S5.pdf]

Table S5. Kirby-Bauer disk-diffusion test results and antimicrobial resistance genes detected among SDSE isolates.

Strain	Antimicrobial						AMR genes
	Penicillin	Vancomycin	Gentamycin	Tetracycline	Clindamycin	Erythromycin	
NSDE00029	S	S	S	S	R	R	*ermA*
NSDE00030	S	S	S	S	S	S	
NSDE00031	S	S	S	S	S	S	
NSDE00033	S	S	S	S	R	R	*ermA, ermT*
NSDE00034	S	S	S	S	S	S	
NSDE00035	S	S	S	S	S	S	
NSDE00036	S	S	S	S	S	S	
NSDE00038	S	S	S	S	S	S	
NSDE00040	S	S	S	S	S	S	
NSDE00041	S	S	S	S	S	S	
NSDE00043	S	S	S	I	S	R	*tetM, msrD*
NSDE00044	S	S	S	S	S	S	
NSDE00045	S	S	S	S	S	S	
NSDE00046	S	S	S	S	induc. R[a]	R	*ermA*
NSDE00047	S	S	S	S	S	S	
NSDE00048	S	S	S	S	S	S	
NSDE00049	S	S	S	S	S	S	
NSDE00051	S	S	S	S	S	S	
NSDE00052	S	S	S	S	R	R	*ermB*
NSDE00053	S	S	S	S	S	S	
NSDE00054	S	S	S	R	R	R	*aadE, tetO, ermB*
NSDE00055	S	S	S	R	R	R	*aadE, tetO, ermB*
NSDE00056	S	S	S	S	S	S	
NSDE00057	S	S	S	S	induc. R	R	*ermA*
NSDE00058	S	S	S	S	S	S	
NSDE00061	S	S	S	S	S	S	
NSDE00065	S	S	S	S	S	S	
NSDE00066	S	S	S	S	S	S	*ermT*
NSDE00068	S	S	S	S	S	S	
NSDE00069	S	S	S	S	S	S	
NSDE00070	S	S	S	S	S	S	
NSDE00071	S	S	S	S	S	S	
NSDE00072	S	S	S	S	S	S	
NSDE00073	S	S	S	S	S	S	
NSDE00075	S	S	S	S	S	S	*ermT*
NSDE00076	S	S	S	R	induc. R	R	*ermA, ermT*
NSDE00078	S	S	S	S	S	S	*ermT*
NSDE00080	S	S	S	S	S	S	*ermT*
NSDE00081	S	S	S	S	S	S	
NSDE00083	S	S	S	S	S	S	

Strain	Antimicrobial						AMR genes
	Penicillin	Vancomycin	Gentamycin	Tetracycline	Clindamycin	Erythromycin	
NSDE00084	S	S	S	S	induc. R	R	*tetM, ermA*
NSDE00085	S	S	S	S	S	S	
NSDE00086	S	S	S	S	S	S	*ermT*
NSDE00088	S	S	S	S	S	S	*ermT*
NSDE00089	S	S	S	S	S	S	*ermT*
NSDE00090	S	S	S	S	S	S	*ermT*
NSDE00091	S	S	S	S	S	S	
NSDE00094	S	S	S	S	S	S	*ermT*
NSDE00095	S	S	S	S	S	S	*ermT*

[a]Inducible resistance in the D-test.
