# Supplementary material for: Dominance of the ST20 stG62647 Lineage Among Invasive Streptococcus dysgalactiae subsp. equisimilis Infections in Toronto, Canada
Source: Microorganisms. 2026 Apr 14;14(4):878. doi: 10.3390/microorganisms14040878 (PMC13119170; doi:10.3390/microorganisms14040878)
Supplement: Supplementary file 1 [file Figure-S2.pdf › Figure-S2.pdf]

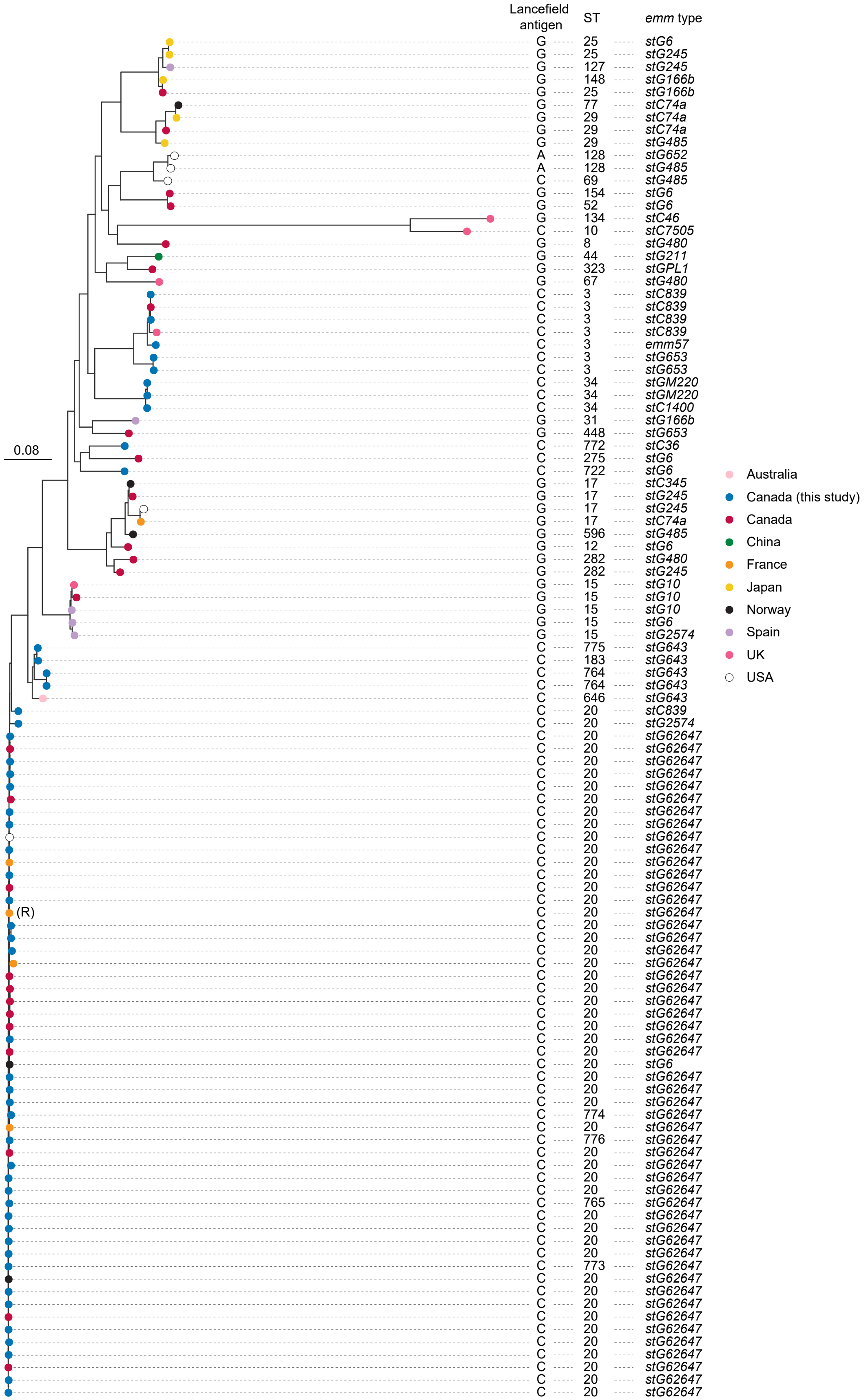

Figure S2. Inferred genetic relationships among SDSE isolates from Toronto and global regions**.** Illustrated is a maximum likelihood phylogenetic tree constructed from 55,842 non-redundant core-genome SNPs identified relative to the genome of the French ST20 *emm*-type *stG62647* reference strain MGCS36044, which is also included in the tree and marked as 'R'. Included in the analysis are the 50 SDSE isolates from metropolitan Toronto (2017, 2018-2022) and 57 additional SDSE isolates from diverse geographic regions including Manitoba, Canada, as well as the United States, France, Norway, Spain, China, the United Kingdom, Australia, and Japan. The geographic origin of each isolate is indicated by the color of the tip branches. Annotations on the tree indicate the Lancefield carbohydrate antigen, multilocus sequence typing, and *emm*-typing results for each isolate.
